# Goal-concordant outcomes in twin pregnancies: impact of chorionicity, fetal weight discordance, and fetal surgery in a 12-year prospective cohort study

**DOI:** 10.3389/fmed.2026.1726115

**Published:** 2026-02-10

**Authors:** Lubomír Hašlík, Ladislav Krofta, Petra Hanulíková, Matúš Halaj, Barbora Beňová, Jiří Hanáček

**Affiliations:** 1 Institute for the Health of Mother and Child, Prague, Czechia; 2 3rd Medical Faculty, Charles University, Prague, Czechia

**Keywords:** birth weight, dichorionic twins, fetal surgery, goal-concordant outcome, intrauterine intervention, monochorionic twins, premature birth, twin pregnancy

## Abstract

**Objective:**

The objective of the study was to introduce a goal-concordant outcome framework and evaluate the independent effects of chorionicity, fetal weight discordance ≥25%, and intrauterine fetal surgery on survival, timing of delivery, and neonatal growth in twin pregnancies.

**Methods:**

This prospective cohort study included 1,860 twin pregnancies delivered ≥24 weeks at a national referral center between 2012 and 2023: 1,097 pregnancies were dichorionic (DC), 559 were monochorionic without intervention (MC), and 204 were monochorionic with intrauterine surgery (MCS; 167 fetoscopic laser procedures and 37 selective reductions). The primary outcome was goal-concordant survival (GCS), defined as dual survival in DC and MC pregnancies, dual survival after laser therapy, or intended singleton survival after selective reduction. The secondary outcomes included goal-concordant week (GCW), defined as delivery within guideline-based gestational age windows, and goal-concordant weight (GCWt), defined as birthweight ≥1,500 g in all surviving neonates. Independent predictors of failure to achieve each outcome were assessed using a multivariable logistic regression analysis.

**Results:**

GCS was achieved in 99.0% of the DC, 98.4% of the MC, and 79.9% of the MCS pregnancies, including 78.4% after laser therapy and 86.5% after selective reduction. In a multivariable analysis, MCS was the strongest independent predictor of failure to achieve GCS (OR: 21.2, 95% CI: 10.5–42.6, *p* < 0.001), while fetal weight discordance ≥25% remained an additional independent risk factor (OR: 2.0, 95% CI: 1.1–3.6, *p* = 0.018). GCW was achieved in 52.1% of the DC, 54.2% of the MC, and 37.9% of the MCS pregnancies. Failure to achieve GCW was independently associated with fetal weight discordance ≥25% in DC and MC twins (OR: 2.9–3.1, *p* ≤ 0.002) and with MCS even in the absence of significant discordance (OR: 1.66, *p* = 0.006). GCWt was achieved in 82.9% of the DC, 69.4% of the MC, and 52.3% of the MCS pregnancies. Failure to achieve GCWt was most strongly associated with fetal weight discordance ≥25% in DC twins (OR 7.2, *p* < 0.001). Across chorionicity groups, pregnancies with fetal weight discordance ≥25% delivered 12–19 days earlier and had neonatal birthweights reduced by approximately 680–800 g.

**Conclusion:**

Chorionicity, fetal weight discordance, and intrauterine fetal surgery independently and differentially affect survival, delivery timing, and neonatal growth. Goal-concordant outcomes provide a clinically intuitive, guideline-aligned benchmark for counseling and outcome comparison in twin pregnancies.

## Introduction

Twin pregnancies account for a disproportionate share of perinatal morbidity and mortality when compared with singleton gestations, despite representing a relatively small fraction of all births (1, 2). This excess risk is driven by a combination of maternal complications, preterm birth, fetal growth disturbances, and perinatal mortality, all of which place twin pregnancies among the most complex scenarios in contemporary obstetric care (3–10). As a result, counseling parents and planning management require not only accurate risk stratification but also outcome measures that reflect realistic clinical goals.

Among the determinants of outcome, chorionicity plays a central role. Dichorionic (DC) twin pregnancies generally follow a more favorable course, whereas monochorionic (MC) twins are uniquely exposed to complications arising from shared placental circulation and intertwin vascular anastomoses (1, 7, 11, 12). These placental characteristics underlie conditions such as twin-to-twin transfusion syndrome (TTTS), twin anemia–polycythemia sequence (TAPS), twin reversed arterial perfusion (TRAP), and selective fetal growth restriction (sFGR), all of which substantially increase the risk of adverse perinatal outcomes (13, 14). Consequently, monochorionic pregnancies require closer surveillance and, in selected cases, invasive fetal therapy (12).

Fetal growth imbalance represents another significant factor influencing outcomes in twin pregnancies (15). A birthweight discordance of 25% or more has consistently been associated with earlier delivery, increased neonatal morbidity, and higher perinatal mortality (16). Growth discordance reflects unequal placental sharing and chronic intertwin imbalance, providing prognostic information that extends beyond gestational age or absolute birthweight alone (17, 18).

In the most complex MC pregnancies, intrauterine interventions such as fetoscopic laser ablation, selective fetal reduction, or radiofrequency ablation are increasingly used to manage life-threatening complications (13, 14, 19–21). Outcomes in these pregnancies are commonly reported using traditional endpoints such as double survival or median gestational age at delivery. While informative, such measures do not always align with the intended clinical goals of treatment, particularly when the objective is to protect a single fetus rather than to ensure the survival of both.

The concept of evaluating outcomes against predefined clinical goals has been explored in other areas of medicine, where treatment success is defined by alignment between outcomes and the intended goals of care rather than by a single biological endpoint (22, 23). To the best of our knowledge, such an approach has not been systematically applied in perinatal or fetal medicine, although clinical objectives in twin pregnancies may vary substantially depending on chorionicity, placental pathology, and management strategy.

In this study, we used a goal-concordant outcome framework to a large, prospectively collected cohort of twin pregnancies. By defining outcomes according to intended clinical goals and examining survival, delivery timing, and neonatal growth simultaneously, we aimed to assess how chorionicity, fetal weight discordance, and intrauterine fetal surgery relate, independently and in combination, to clinically meaningful outcomes.

## Methods

### Study design and population

The study design aimed to assess whether contemporary management strategies in twin pregnancies lead to the achievement of predefined clinical objectives rather than the isolated per-fetus outcomes. Accordingly, this single-center prospective observational cohort study included all twin pregnancies delivered at ≥24 + 0 weeks of gestation at the tertiary perinatal center of the Institute for the Health of Mother and Child (IHMC) between 1 January 2012 and 31 December 2023.

All consecutive eligible twin pregnancies were included to minimize selection bias.

During the study period, 1,860 twin pregnancies met the inclusion criteria, comprising 1,097 dichorionic (DC) and 763 monochorionic (MC) twin pregnancies. Among the MC pregnancies, 204 underwent intrauterine surgery (MCS). All pregnancies were monitored and delivered at the IHMC according to the standardized institutional protocols.

### Data collection and ethics

Prospective data capture was chosen to minimize information bias and ensure uniform outcome assessment. Prospective data collection followed a predefined protocol approved by the institutional ethics committee (reference No. 3/5/5). The data of all twin pregnancies monitored and delivered at the IHMC were stored in the hospital’s FONS AKORD computer system. Data were entered into a dedicated electronic database with annual audits to ensure completeness and consistency. Records with missing or unverifiable data were excluded from the analysis.

### Primary outcome

The primary outcome was selected to reflect whether the predefined clinical objective of each pregnancy was achieved. The primary outcome was goal-concordant survival (GCS), defined according to the intended clinical pathway:

1. Survival of both DC and MC twins without intrauterine intervention.
2. Dual survival following fetoscopic laser surgery for twin–twin transfusion syndrome (TTTS) or twin anemia–polycythemia sequence (TAPS).
3. Survival of the intended singleton after selective fetal reduction using bipolar cord occlusion (BPO) or radiofrequency ablation (RFA).

By definition, GCS integrates treatment intent with final survival, aligning outcome assessment with real-world clinical decision-making and parental counseling.

### Secondary outcomes

The secondary outcomes were designed to capture whether survival was achieved under optimal timing and growth conditions.

Goal-concordant week (GCW) was defined as delivery within a predefined optimal gestational age window, adapted to chorionicity and treatment pathway.

Gestational age windows were derived from contemporary international guideline recommendations for twin pregnancies and fetal therapy cohorts to balance prematurity-related risk against intrauterine disease progression (24–26).

The target windows were:

- DC twins: 37 + 0 to 39 + 0 weeks
- MC twins: 35 + 0 to 37 + 0 weeks
- MCS after fetoscopic laser surgery: 34 + 0 to 37 + 0 weeks
- MCS after selective reduction: 37 + 0 to 40 + 0 weeks

Goal-concordant weight (GCWt) was defined as a birthweight ≥1,500 g in all surviving neonates at delivery; in pregnancies after selective reduction, GCWt was assessed for the intended survivor only. The 1,500 g threshold was chosen to represent the established boundary between very low and low birth weight, a clinically meaningful transition associated with a substantial reduction in severe neonatal morbidity and healthcare resource utilization.

To provide additional clinical granularity, an exploratory analysis of neonates with birth weight ≥2,000 g was included. This threshold reflects a level of neonatal maturity associated with markedly lower morbidity and reduced need for intensive care and was intended to complement, not redefine, the primary GCWt outcome.

Conventional outcomes included gestational age at delivery, birth weight of the larger and smaller twin, and absolute intertwin birth weight difference.

Eligibility criteria were structured to create a homogeneous cohort in which outcomes could be attributed primarily to chorionicity, growth patterns, and intrauterine intervention.

The inclusion criteria were as follows:

- DC and MC twin pregnancies delivered at ≥24 + 0 weeks of gestation without diagnosed genetic abnormalities
- MC twin pregnancies with structural anomalies managed by intrauterine selective reduction (BPO and RFA)

The exclusion criteria were as follows:

- DC or MC pregnancies complicated by spontaneous single or double intrauterine demise before 24 weeks of gestation or diagnosed genetic abnormalities.
- DC pregnancies with structural anomalies, elective selective reduction performed before 12 weeks of gestation, or selective reduction for genetic or structural anomalies before 24 weeks of gestation.
- Fetuses with postnatally diagnosed but prenatally unrecognized genetic or structural anomalies.
- Pregnancies lacking complete data for primary or secondary outcomes.

### Ultrasound surveillance and pregnancy management

A standardized ultrasound surveillance protocol was used to ensure consistent exposure and follow-up across the cohort. Chorionicity was determined in the first trimester using standard sonographic criteria, including assessment of the number of gestational sacs, lambda sign, membrane thickness, and crown–rump length-based gestational age dating. First-trimester image documentation was retained as a reference throughout the pregnancy. Routine ultrasound surveillance was performed every 2 weeks, with an increased frequency in complicated pregnancies. Each examination included a systematic assessment of fetal growth, amniotic fluid volume, Doppler velocimetry of the umbilical and middle cerebral arteries, and evaluation of the fetal venous system. All ultrasound data were stored using Astraia Nexus software (version 29.1.2).

Planned delivery timing was predefined to reduce variability related to the preferences of individual physicians. Targeted delivery was planned at 38 + 0–39 + 0 weeks for DC twins and 36 + 0–37 + 0 weeks for MC twins (34 + 0–37 + 0 weeks after fetoscopic laser surgery).

Iatrogenic delivery was defined *a priori* as delivery for maternal or fetal indications not meeting criteria for spontaneous preterm labor or preterm prelabor rupture of membranes (PPROM).

### Definitions of growth restriction and discordance

The definitions of fetal growth restriction were aligned with contemporaneous consensus standards to preserve temporal validity throughout the study period. Diagnostic criteria for selective fetal growth restriction (sFGR) evolved:

- Before 1 February 2019, sFGR was diagnosed if any of the following were present: intertwin weight discordance ≥25%, estimated fetal weight of the smaller twin <10th percentile, or umbilical artery pulsatility index ≥90th percentile in the smaller twin.
- From 1 February 2019 onward, sFGR was defined according to the Delphi consensus criteria.

Birth weight discordance was defined postnatally and uniformly as ≥25% using the formula [(BW larger − BW smaller) / BW larger] × 100 to allow consistent outcome classification.

### Intrauterine procedures

All intrauterine interventions were performed in accordance with internationally accepted diagnostic criteria and technical standards. TTTS was staged according to the Quintero classification (27). TAPS was diagnosed according to the Delphi consensus criteria (28); before their publication, the diagnosis was based on an intertwin MCA-PSV difference of ≥0.7 MoM.

Fetoscopic laser ablation of placental vascular anastomoses was performed using an Nd: YAG laser (20–25 W) with a 10-Fr and 3.3-mm catheter and Karl Storz fetoscopes between 16 + 4 and 30 + 1 weeks. The Solomon technique was used in all cases. Of the 167 analyzed cases, 6 (3.6%) were classified as stage I, 29 (17.4%) as stage II, 116 (69.5%) as stage III, and 16 (9.6%) as stage IV. The analyzed interventions were counted only for TTTS or TAPS diagnoses; laser ablations performed for other diagnoses (sFGR and congenital anomalies) were excluded from the analysis.

BPO was performed for severe congenital anomalies or early severe growth restriction using bipolar forceps (20–40 W) between 16 + 2 and 26 + 3 weeks.

RFA was performed exclusively for twin reversed arterial perfusion sequence using a RITA 1500X generator (Angiodynamics, United States) with an output of 1–125 W and a frequency of 460 kHz. Within 15 + 6 and 23 + 0 weeks, StarBurst needles were used for target lesions with a diameter of 1–5 cm and a catheter size of 6.4 Fr.

### Statistical analysis

Statistical methods were selected to reflect the paired nature of twin data and the hierarchical structure of goal-concordant outcomes. Statistical analyses were performed using IBM SPSS Statistics version 29.0. Non-parametric tests for numerical variables and tests of independence in contingency tables were used to evaluate the individual variables (Tables 1–4). Odds ratios were estimated using a multivariate logistic regression analysis, including possible interactions (Table 2). When statistically significant, interaction terms between chorionicity, fetal weight discordance ≥25%, and intrauterine intervention were tested and included in the final models. The multivariate analysis was based on the two-way analysis of variance (ANOVA) (Table 3). Logistic regression models included maternal age, conception type (assisted reproductive technology (ART) vs. spontaneous), parity, and gestational age at diagnosis as covariates.

**Table 1:** Basic demographic parameters and selected parameters related to pregnancy.

Parameter	DC twins	MC twins	MCS twins	*p*
	N-1,097 (59%)	N-559 (30%)	N-204 (11%)	
Median mother age at birth (years)	34 (30.5–37)	32 (29–36)	31 (28–35)	**<0.001** ^§^
Parity				
Para 0	443 (40.4%)	193 (34.5%)	82 (40.2%)	0.060^‡^
Type of conception				
Assisted Reproduction (IVF/ICSI)	399 (36.4%)	105 (18.8%)	22 (10.8%)	**<0.001** ^‡^
Spontaneous	698 (63.6%)	454 (81.2%)	182 (89.2%)	
Delivery median (week)	37 (34–38)	35 (33–36)	32 (29–35)	**<0.001** ^§^
Birth weight median (g)				
Larger neonate (g)	2,520 (2030–2,850)	2080 (1675–2,430)	1715 (1210–2262.5)	**<0.001** ^§^
Smaller neonate (g)	2,240 (1770–2,550)	1820 (1380–2,230)	1,480 (990–1840)	**<0.001** ^§^
Type of delivery				
Caesarean section	1,037 (94.5%)	548 (98%)	153 (74.8%)	**<0.001** ^‡^
Vaginal birth	60 (5.5%)	11 (2%)	27 (13.4%)	
IUFD	0 (0%)	0 (0%)	24 (11.9%)	
Number of newborns				
IUFD both fetuses	0 (0%)	6 (1.1%)	26 (12.7%)	**<0.001** ^‡^
IUFD one fetus	11 (1%)	3 (0.5%)	47 (23%)	
Double survival	1,086 (99%)	550 (98.4%)	131 (64.2%)	
Gestational age				
<= 26	26 (2.4%)	13 (2.3%)	32 (15.8%)	**<0.001** ^*^
27–28	28 (2.6%)	18 (3.2%)	12 (5.9%)	
29–32	127 (11.6%)	96 (17.2%)	59 (29.1%)	
33–36	312 (28.4%)	304 (54.4%)	75 (36.5%)	
37	604 (55.1%)	128 (22.9%)	26 (12.8%)	
Delivery indication				
Spontaneous uterine contractions	220 (20.1%)	77 (13.8%)	19 (9.2%)	**<0.001** ^*^
Iatrogenic	212 (19.3%)	47 (8.4%)	55 (27.2%)	
PPROM	665 (60.6%)	435 (77.8%)	130 (63.6%)	

Numerical variables are reported as median (lower quartile–upper quartile). Categorical variables are reported as frequency (*n*) and percentage (%). The bold emphasized variables show statistically significant (at a 5% significance level) differences in proportion between groups. Statistics: §Kruskal-Wallis, ‡Fisher Exact test; *Chi-Square test with Monte Carlo simulation on 10,000 samples.

Bold values indicate statistically significant differences at the 5% level.

**Table 2:** Odds ratio of goal concordant survival for monochorionicity with and without interventions vs. dichorionicity and weight discrepancy over 25% vs. under 25%.

Adverse goal concordant survival	OR with 95% CI	*p*
MC no interventions	1.513 (0.621–3.684)	0.362*
MC with interventions	21.15 (10.497–42.615)	<0.001*
Weight discrepancy ≥ 25%	2.022 (1.126–3.631)	0.018*

Statistics: *Wald test from logistic regression.

Bold values indicate statistically significant differences at the 5% level.

**Table 3:** The results of the two-way ANOVA, categorized by twin type and intervention level, include weight discrepancy.

Parameter	Weight discrepancy < 25%	Weight discrepancy ≥ 25%	*p*	
Birth weight of larger neonate [g]*				
DC twins	2,422 (2384.4–2459.6)	2185.5 (2072.2–2298.7)	**<0.001** ^c^	**<0.001** ^g^
MC twins	2107.3 (2052–2162.7)	1662.8 (1542.1–1783.5)	**<0.001** ^d^	
MCS twins	1753 (1644.6–1861.4)	1833.5 (1674–1992.9)	0.511^e^	
*p*	**<0.001** ^a^	**<0.001** ^b^	**<0.001** ^h^	
	**<0.001** ^f^			
Birth weight of smaller neonate [g]*				
DC twins	2188.8 (2155.4–2222.2)	1508.3 (1406.2–1610.4)	**<0.001** ^a^	**<0.001** ^f^
MC twins	1900.1 (1851.1–1949.2)	1101.7 (993.7–1209.6)	**<0.001** ^b^	
MCS twins	1537.5 (1432.3–1642.7)	1176.8 (979–1374.6)	**<0.001** ^h^	
*p*	**<0.001** ^c^	**<0.001** ^d^	**<0.001** ^f^	
	**<0.001** ^c^			
Weight difference [g]*				
DC twins	237.4 (226.6–248.3)	702.2 (669.6–734.9)	**<0.001** ^a^	**<0.001** ^f^
MC twins	208.1 (192.3–224)	570 (535.5–604.6)	**<0.001** ^b^	
MCS twins	126.9 (85.3–168.5)	124 (−28.3–276.3)	0.951^h^	
*p*	**<0.001** ^c^	**<0.001** ^d^	**0.002** ^f^	
	**<0.001** ^c^			
Delivery week*				
DC twins	35 + 5 (35 + 4–35 + 7)	34 + 0 (33 + 3–34 + 5)	**<0.001** ^a^	**<0.001** ^f^
MC twins	34 + 3 (34 + 1–34 + 5)	31 + 5 (31 + 0–32 + 2)	**<0.001** ^b^	
MCS twins	31 + 4 (30 + 7–32 + 1)	31 + 3 (30 + 4–32 + 2)	0.911^h^	
*p*	**<0.001** ^c^	**<0.001** ^d^	**0.002** ^f^	
	**<0.001** ^c^			

Expected values with their 95% CI are reported alongside *p*-values testing particular hypotheses in the model. The bold emphasized variables show statistically significant (at a 5% significance level) differences in proportion between groups. Dichorionic (DC), monochorionic (MC), monochorionic with intrauterine surgery (MCS). Tested effects: ^a^Test of the effect of twin type on weight discrepancy under 25%. ^b^Test of the effect of twin type at a weight discrepancy of 25% and over. ^c^Test of the effect of weight discrepancy at DC twins. ^d^Test of the effect of weight discrepancy at MC twins. ^e^Test of the effect of weight discrepancy at MCS twins. ^f^Test of the effect of twin type at both levels of weight discrepancy. ^g^Test of the effect of weight discrepancy at all levels of twin type. ^h^Test of interaction of twin type and weight discrepancy. Statistics: *F test from Two-way ANOVA with robust standard errors.

Bold values indicate statistically significant differences at the 5% level.

**Table 4:** Selected parameters related to the pregnancy of twins with intervention according to intervention type.

Parameter	LASER	BO or RFA/TRAP	*p*
	N-167 (81.9%)	N-37 (18.1%)	
Median mother age at birth (years)	34 (30.5–37)	32 (29–36)	0.345^§^
Parity			
Para 0	63 (37.7%)	19 (51.4%)	0.129^‡^
Type of conception			
Assisted Reproduction (IVF/ICSI)	17 (10.2%)	5 (13.5%)	0.561^‡^
Spontaneous	150 (89.8%)	32 (86.5%)	
Delivery median (week)	32 (29–35)	36 (31–38 + 5)	**<0.001** ^§^
Intervention to delivery [day]	69 (39–90)	94 (56.75–109.5)	**0.007** ^§^
Birth weight median of larger neonate [g]	1,600 (1200–2127.5)	2,490 (1797.5–2947.5)	**<0.001** ^§^
Birth weight median of smaller neonate [g]	1,480 (990–1840)	–	–
Type of delivery			
Caesarean section	137 (82.0%)	16 (43.2%)	**<0.001** ^‡^
Vaginal birth	11 (6.6%)	16 (43.2%)	
IUFD	19 (11.4%)	5 (13.6%)	
Number of newborns			
IUFD both fetuses	21 (12.6%)	5 (13.5%)	**<0.001** ^‡^
IUFD one fetus	15 (9%)	32 (86.5%)	
Double survival	131 (78.4%)	0 (0%)	
Gestational age			
<= 26	27 (16.2%)	5 (13.9%)	<0.001*
27–28	11 (6.6%)	1 (2.8%)	
29–32	55 (32.9%)	4 (11.1%)	
33–36	64 (38.3%)	10 (27.8%)	
37	10 (6%)	17 (44.4%)	
Delivery indication			
Spontaneous uterine contractions	16 (9.3%)	3 (9.1%)	0.357*
Iatrogenic	49 (29.1%)	5 (13.6%)	
PPROM	103 (61.6%)	29 (77.3%)	

Numerical variables are reported as median (lower quartile–upper quartile). Categorical variables are reported as frequency (n) and percentage (%). The bold emphasized variables show statistically significant (at a 5% significance level) differences in proportion between groups. Statistics: §Mann–Whitney test, ‡Exact Fisher Test, *Chi-Square test with Monte Carlo simulation on 10,000 samples.

Bold values indicate statistically significant differences at the 5% level.

## Results

### Study population

Between January 2012 and December 2023, a total of 1,860 twin pregnancies were included in this study, as detailed in Table 1. The cohort consisted of 1,097 dichorionic (DC) pregnancies, 559 monochorionic pregnancies without intervention (MC), and 204 monochorionic pregnancies with intrauterine surgery (MCS). Within the MCS group, 167 pregnancies underwent fetoscopic laser ablation for TTTS/TAPS, while 37 underwent selective reductions (BPO and RFA). A weight discrepancy of ≥ 25% was observed in 109 (9.9%) cases in the DC group, 98 (17.4%) cases in the MC group, and 77 (37.7%) cases in the MCS group. Maternal age and proportion of assisted reproduction were highest in the DC group.

### Primary outcome: goal-concordant survival

**Figure 1:**
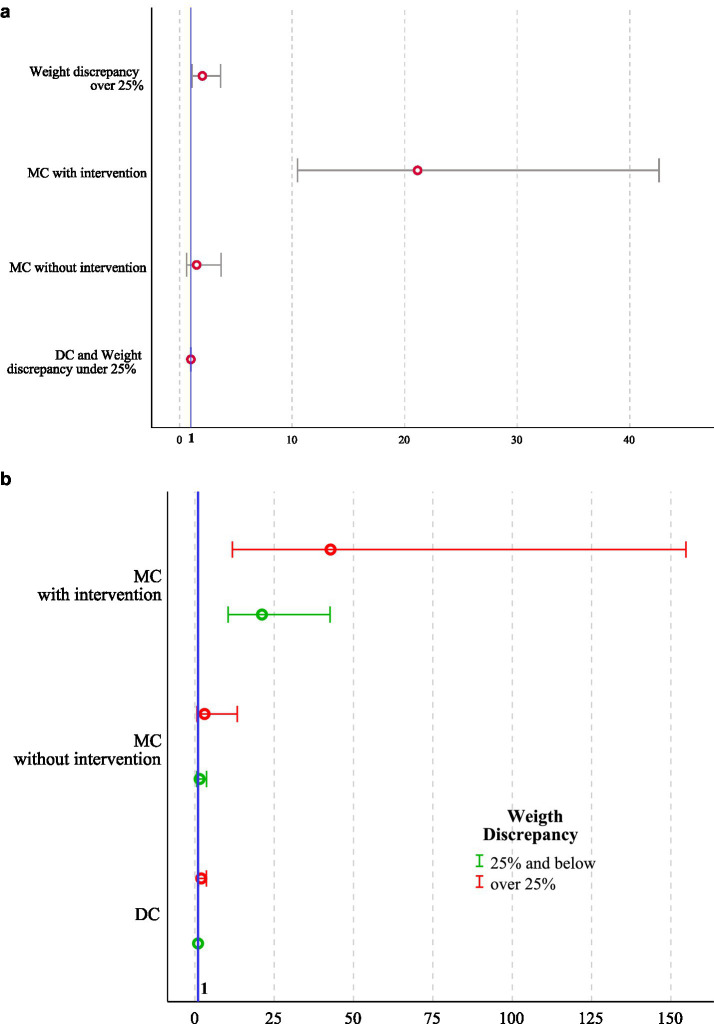
**(a)** Plot presenting univariable OR with 95% CI for failure to achieve GCS. Odds ratios are shown for the individual effects of chorionicity (MC vs. DC), intrauterine intervention (yes vs. no), and fetal weight discordance (≥25% vs. <25%), with DC twin pregnancies with fetal weight discordance <25% as the reference category (OR = 1). **(b)** Adjusted OR with 95% CI for failure to achieve GCS across all feasible combinations of chorionicity, intrauterine intervention, and fetal weight discordance (≥25% vs. <25%). ORs were estimated using a multivariable logistic regression analysis, with DC twin pregnancies with fetal weight discordance <25% as the reference category (OR = 1). The number of twin pregnancies was 1,860. OR, Odds ratio; CI, Confidence interval; DC, Dichorionic twins; MC, Monochorionic twins without intervention; MCS, Monochorionic twins with intrauterine surgery; GCS, Goal-concordant survival.

GCS was achieved in 1086 (99%) DC, 550 (98.4%) MC, and 163 (79.9%) MCS pregnancies (*p* < 0.001). Within the MCS subgroup, dual survival after laser was achieved in 131 cases (78.4%), while intended singleton survival after selective reduction was achieved in 32 cases (86.5%). A multivariable logistic regression analysis identified both monochorionicity with intervention (OR 21.2, 95% CI 10.5–42.6, *p* < 0.001) and prenatal weight discrepancy ≥ 25% (OR 2.02, 95% CI 1.13–3.63, *p* = 0.018) as independent predictors of GCS failure (Table 2; Figures 1a,b).

### Secondary outcomes: goal-concordant week and goal-concordant weight

**Figure 2:**
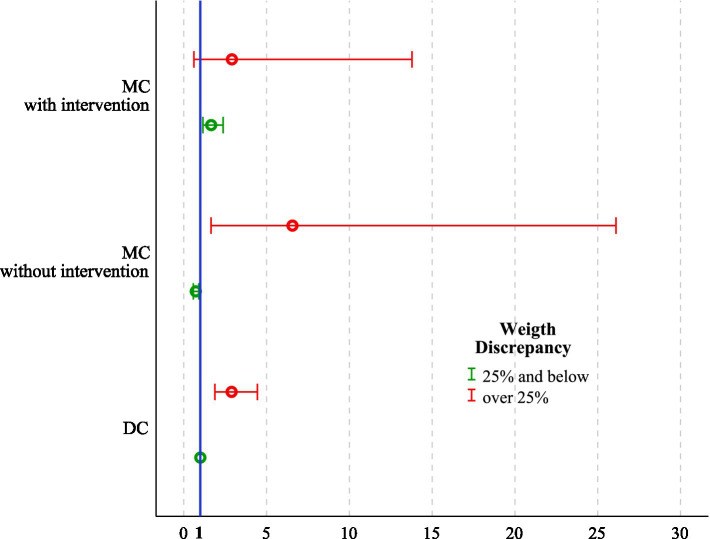
Adjusted OR with 95% CI for failure to achieve GCW across all feasible combinations of chorionicity, intrauterine intervention, and fetal weight discordance (≥25% vs. <25%). ORs were estimated using a multivariable logistic regression analysis, with DC twin pregnancies with fetal weight discordance <25% as the reference category (OR = 1). The number of twin pregnancies was 1,860. OR, Odds ratio; CI, Confidence interval; DC, Dichorionic twins; MC, Monochorionic twins without intervention; MCS, Monochorionic twins with intrauterine surgery; GCW, Goal-concordant week.

The median gestational age at delivery was 37 weeks in DC, 35 weeks in MC, and 32 weeks in MCS pregnancies (*p* < 0.001). GCW was achieved in 571 (52.1%) DC, 303 (54.2%) MC, and 78 (37.9%) MCS pregnancies (*p* < 0.001). Among surgical cases, GCW achievement was noted in 62 cases (37.1%) after laser and 16 cases (43.2%) after selective reductions. A logistic regression analysis revealed that MC twins with a birthweight discrepancy ≥ 25% (OR 3.13, 95% CI 1.52–6.46, *p* = 0.002), a weight discrepancy ≥ 25% in DC twins (OR 2.89, 95% CI 1.88–4.44, *p* < 0.001), and MCS even in the absence of significant discrepancy (OR 1.66, 95% CI 1.16–2.37, *p* = 0.006) were independent risk factors for not achieving the goal-concordant week of delivery (Table 2; Figure 2).

GCWt (all survivors ≥ 1,500 g) was achieved in 909 (82.9%) DC, 387 (69.4%) MC, and 107 (52.3%) MCS pregnancies (*p* < 0.001). In the surgical subgroup, rates were 46.5% after laser and 78.1% after reduction. Using the exploratory ≥2,000 g threshold, GCWt achievement declined across all groups. Within the MCS cohort, lower rates after laser therapy contrasted with higher apparent achievement after selective reduction, reflecting differences in delivery timing and population at risk rather than a true biological advantage (Table 4).

**Figure 3:**
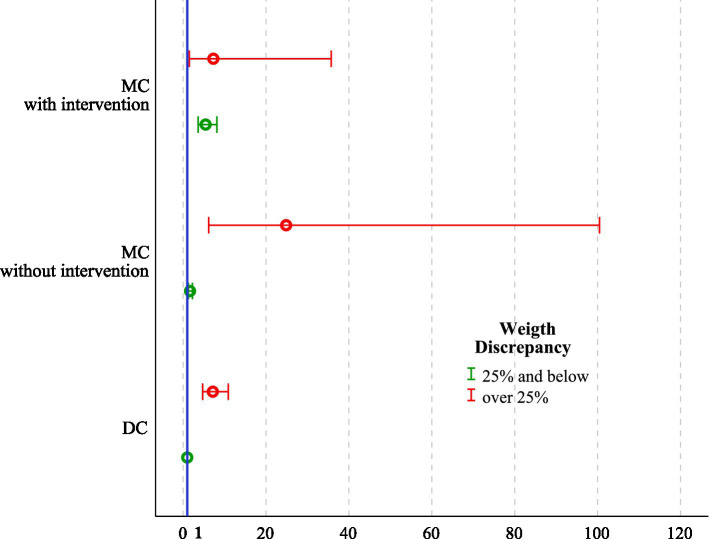
Adjusted OR with 95% CI for failure to achieve GCWt across all feasible combinations of chorionicity, intrauterine intervention, and fetal weight discordance (≥25% vs. <25%). ORs were estimated using a multivariable logistic regression analysis, with dichorionic twin pregnancies with fetal weight discordance <25% as the reference category (OR = 1). The number of twin pregnancies was 1,860. OR, Odds ratio; CI, Confidence interval; DC, Dichorionic twins; MC, Monochorionic twins without intervention; MCS, Monochorionic twins with intrauterine surgery; GCWt, Goal-concordant weight.

The logistic regression analysis revealed that weight discrepancy ≥ 25% in DC twins (OR 7.17, 95% CI 4.72–10.90, *p* = 0.037), MCS with concordant weights (OR 5.44, 95% CI 3.63–8.15, *p* < 0.001), and MC with birthweight discrepancy ≥ 25% (OR 2.07, 95% CI 1.05–4.12, *p* < 0.001) were independent predictors of not achieving goal-concordant neonatal weight. In contrast, MCS and marked discrepancy were associated with a protective effect (OR, 0.19; 95% CI, 0.09–0.40; *p* < 0.001; Table 2; Figure 3). This apparent protective effect reflects the altered population at risk after selective reduction rather than a true biological advantage.

In the subgroup where the difference was equal to or greater than 25%, we noted that birth occurred on average 12 days earlier in DC twins and 19 days earlier in the MC group compared to the cohort with a prenatal weight difference of less than 25% (*p* < 0.001), while no significant shift was observed in the MCS group, where delivery timing was primarily dictated by the intervention itself (Table 3).

### Conventional outcomes

**Figure 4:**
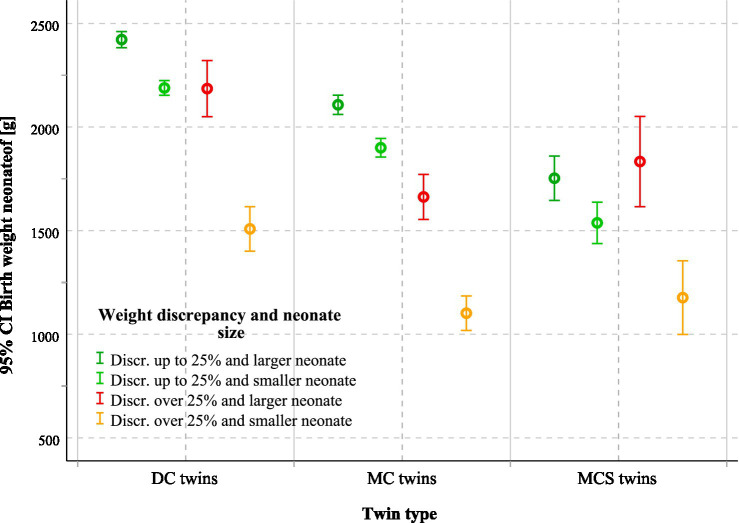
Mean birthweight of larger and smaller neonates according to chorionicity, intrauterine intervention, and fetal weight discordance. The figure shows mean birthweight with 95% CI for larger and smaller neonates in DC, MC, and MCS twins, stratified by fetal weight discordance (≥25% vs. < 25%). The number of twin pregnancies was 1860. DC, Dichorionic twins; MC, Monochorionic twins without intervention; MCS, Monochorionic twins with intrauterine surgery; CI, Confidence interval.

The median birthweight of the larger twin was 2,520 g in DC, 2,080 g in MC, and 1,715 g in MCS pregnancies (*p* < 0.001). For the smaller twin, median weights were 2,240 g, 1,820 g, and 1,480 g, respectively (*p* < 0.001; Table 1). Discordant pregnancies (≥ 25%) showed an additional reduction in the smaller twin’s birth weight of 681 g in the DC group and 798 g in the MC group, whereas in the MCS group, the effect was masked by selective reduction, with survivors averaging 1,520–2,010 g, depending on the intervention (Figure 4). The intertwin birthweight difference was most pronounced in MCS pregnancies, reflecting severe underlying placental pathology. In DC and MC pregnancies, discordance ≥ 25% was consistently associated with greater absolute birthweight gaps and earlier delivery (Table 3).

## Discussion

This study revealed that evaluating twin pregnancies using a goal-concordant framework yields clinically actionable information that cannot be derived from conventional single-endpoint outcome reporting alone, allowing outcomes to be interpreted against clinically expected benchmarks. By sequentially assessing goal-concordant survival (GCS), goal-concordant week of delivery (GCW), and goal-concordant neonatal weight (GCWt), our findings clarify how different pregnancy pathways translate into realistic clinical outcomes.

### Goal-concordant survival

In this cohort, GCS was achieved in more than 98% of the DC and non-intervened MC pregnancies. This finding is fully consistent with large cohort studies and meta-analyses demonstrating that, under contemporary surveillance, survival of both fetuses represents the expected benchmark in the vast majority of uncomplicated twin pregnancies (6, 29). In this context, survival alone has limited discriminatory value for risk stratification or parental counseling in DC and uncomplicated MC twin pregnancies.

In contrast, MC pregnancies requiring intrauterine intervention showed a substantially lower rate of GCS. This finding is consistent with the fetal therapy literature, where survival rates are strongly influenced by disease severity at presentation rather than by the technical success of the procedure itself (13, 21, 26, 27). Importantly, our multivariable analysis revealed that failure to achieve GCS is independently associated not only with intrauterine surgery but also with fetal weight discordance ≥25%. This finding indicates that severe placental imbalance and procedure-related hemodynamic changes remain dominant determinants of survival, even in the presence of advanced fetal therapy.

From a clinical counseling perspective, this distinction is critical. Parents can be informed that fetal intervention often enables survival of the intended fetus, but that survival cannot be guaranteed and is primarily driven by the underlying placental pathology rather than by the procedure alone (30–32). This reframing helps avoid unrealistic expectations and supports more balanced decision-making.

### Goal-concordant week

While survival is frequently reported as the primary outcome, timing of delivery is often underemphasized despite its major impact on neonatal morbidity. In our cohort, approximately half of the DC and non-intervened MC pregnancies achieved delivery within guideline-recommended gestational age windows, and achievement was even lower in surgically managed MC pregnancies.

These results extend prior reports that describe earlier median gestational age in MC and growth-discordant twins by demonstrating that a large proportion of pregnancies fail to reach an optimal delivery window, even when survival is achieved (17–19, 33). Fetal weight discordance ≥25% emerged as a strong and consistent predictor of GCW failure in DC and MC twins, advancing delivery by approximately 2 to 3 weeks. This aligns with existing literature linking discordance to placental insufficiency, obstetric intervention, and spontaneous preterm birth (15, 34, 35).

Clinically, GCW provides a practical framework for counseling that shifts the discussion from abstract risks of prematurity to a concrete question: *What is the likelihood that delivery will occur within the recommended window?* This is particularly valuable when balancing expectant management against intervention in borderline cases.

### Goal-concordant weight

Neonatal weight represents a cumulative outcome of gestational duration and intrauterine growth and is strongly associated with neonatal morbidity (8, 36, 37). The progressive decline in GCWt achievement from DC to MC to surgically managed pregnancies mirrors patterns described in studies of placental sharing and selective growth restriction (7, 8, 35, 38). Our data further demonstrate that fetal weight discordance ≥25% is a powerful independent predictor of failure to achieve GCWt in both DC and MC twins, reinforcing its prognostic relevance beyond gestational age alone.

In surgically managed pregnancies, interpretation of GCWt requires careful contextualization. Selective reduction alters the population at risk and may result in apparently favorable weight-based outcomes by enabling later delivery and higher birth weights compared with conservative management. This underscores a limitation of conventional growth reporting and supports the use of goal-concordant metrics (39).

For clinicians, GCWt helps translate abstract growth data into meaningful counseling regarding expected neonatal conditions and postnatal care needs. For parents, it provides a clearer understanding that survival may be accompanied by significant growth-related morbidity, particularly in the presence of marked discordance.

### Integrated clinical implications

Taken together, these results revealed that GCS, GCW, and GCWt capture distinct but complementary dimensions of outcome, each of which is relevant to clinical decision-making. Survival alone substantially underestimates the burden of prematurity and impaired growth, particularly in monochorionic and surgically managed pregnancies. Fetal weight discordance ≥25% consistently emerged as a key determinant across all three domains, supporting its role as a central marker for intensified surveillance, individualized counseling, and management planning.

### Strengths and limitations

This study benefits from a prospective design, a large consecutive cohort, standardized management at a national tertiary referral center, and the use of a goal-concordant outcome framework enabling clinically meaningful interpretation.

However, several limitations warrant emphasis. The single-center design in a high-expertise tertiary referral setting limits generalizability and introduces potential referral bias, particularly in MC pregnancies requiring intrauterine surgery, which represent the most severe cases. The definition of sFGR changed during the study period following the Delphi consensus, which may have affected case classification, although sensitivity analyses suggested stable associations. The outcomes were confined to perinatal survival, gestational age at delivery, and birth weight—important but intermediate endpoints—without data on long-term neonatal or neurodevelopmental outcomes. Finally, despite multivariable adjustment, residual confounding from unmeasured maternal, placental, or neonatal factors cannot be excluded. These limitations highlight the need for multicenter validation and long-term follow-up studies, although they do not undermine the internal validity of the findings.

## Conclusion

This study has shown that outcomes in twin pregnancies cannot be adequately characterized by survival alone. Although survival of the intended fetus or fetuses is achieved in the majority of cases, a substantial proportion of pregnancies—particularly monochorionic and surgically managed ones—fail to reach optimal timing of delivery or clinically meaningful neonatal weight.

Three key findings emerge. First, chorionicity, fetal weight discordance ≥25%, and intrauterine fetal surgery independently and differentially affect survival, delivery timing, and neonatal growth. Second, fetal weight discordance ≥25% consistently identifies pregnancies at a high risk of failing multiple clinically relevant goals across all pregnancy pathways. Third, intrauterine interventions enable the achievement of intended survival but do not fully mitigate the adverse impact of severe underlying placental and hemodynamic pathology.

By applying a goal-concordant framework, these results provide clinicians and parents with clear, intention-aligned answers to the questions most often asked during counseling: *What is the realistic chance of survival? Is delivery likely to occur within an optimal gestational window? What neonatal condition can be expected at birth?* The goal-concordant approach thus represents a practical tool for parental counseling, individualized risk stratification, and meaningful comparison of outcomes across diverse twin pregnancy pathways. Future research should focus on multicenter validation of goal-concordant outcomes and on integrating long-term neonatal and neurodevelopmental follow-up.

## Data Availability

The raw data supporting the conclusions of this article will be made available by the authors, without undue reservation.
